# Listening to the student voice to improve educational software

**DOI:** 10.1080/10872981.2017.1345575

**Published:** 2017-07-05

**Authors:** Mari van Wyk, Linda van Ryneveld

**Affiliations:** ^a^ Department of Science, Mathematics and Technology Education, Faculty of Education, University of Pretoria, Pretoria, South Africa

**Keywords:** Student voice, veterinary education, software development, educational software, ease of use, user experience

## Abstract

Academics often develop software for teaching and learning purposes with the best of intentions, only to be disappointed by the low acceptance rate of the software by their students once it is implemented. In this study, the focus is on software that was designed to enable veterinary students to record their clinical skills. A pilot of the software clearly showed that the program had not been received as well as had been anticipated, and therefore the researchers used a group interview and a questionnaire with closed-ended and open-ended questions to obtain the students’ feedback. The open-ended questions were analysed with conceptual content analysis, and themes were identified. Students made valuable suggestions about what they regarded as important considerations when a new software program is introduced. The most important lesson learnt was that students cannot always predict their needs accurately if they are asked for input prior to the development of software. For that reason student input should be obtained on a continuous and regular basis throughout the design and development phases.

## Introduction

One of the important competencies expected of practising veterinarians is the protection of the health and well-being of animals by diagnosing diseases and treating sick and injured animals [1–3]. Therefore, veterinary students need to be empowered with essential knowledge, skills, and mindsets in the course of their professional training [2]. We know from the literature that students expect to be fully equipped with the practical skills required by their profession so that they can adapt easily to the real world of work [4]. As such, universities that offer veterinary education typically strive to provide students with extensive hands-on training in various clinical settings, including academic veterinary hospitals, laboratories, and private or state-owned veterinary practices [5–7]. Such practical exposure gives students an opportunity to learn in a real-life context and has the potential to develop a wide array of skills, including clinical reasoning, communication, and fine motor skills.

At the university where this research was done, veterinary students are exposed to a number of work-integrated learning experiences in the form of 32 clinical rotations that take place in an 18-month period. This practical hands-on learning follows a number of years of studying a wide range of para- and preclinical modules. Whilst it is almost impossible to guarantee standardised case exposure to all students who visit a particular station during their various rotations, the aim is always to ensure that students are adequately prepared to deal with any typical scenario they might encounter on their first day in practice [1].

For exceptional quality veterinary education, clinical training in small groups is essential [8]. Thus, students usually join a particular section or station in groups of eight to ten on a rotational basis. For various reasons not all students can be guaranteed the exact same level of exposure. For example, some illnesses, such as Parvoviral enteritis (cat flu) and Babesiosis (biliary fever), are more prevalent in a particular season, whilst the natural ebb and flow in any practice benefit those students who are attending the specific rotation when an interesting or a rare case is admitted.

In an attempt to get a comprehensive picture of an individual student’s clinical exposure during his/her training, the director of a South African veterinary academic hospital (OVAH) conceptualised and developed a computer-based logging system called Vetbox [9]. This system was intended to replace the rather inefficient, paper-based logging system that had been used in previous years. One of the aims with the introduction of Vetbox was to provide students with an easily accessible online space where they could log the various procedures they were exposed to during their clinical rotations in real time. The online program was designed as a web-based application and although it could be used on a smartphone or tablet, it did not have a customised mobile application user interface or caching abilities. However, the benefits of such a system are self-explanatory and the concept has been internationally acclaimed for its ability to record specific curriculum outcomes [9]. For example, if one of the outcomes is that students should be able to carry out routine diagnostic tests and procedures (such as haematology, basic clinical pathology, basic imaging) in order to make a diagnosis, the online program should provide them with the opportunity to log the specific procedures that show that certain skills are mastered against a case that they were involved in.

It is a well-documented fact that today’s Generation Y students regard themselves as active participants with a strong desire to shape their own learning experiences [10]. Keiller and Inglis-Jassiem [11] support this notion and state that when academics plan to implement new innovations and technology in teaching and learning, they should consider the students’ voice, especially in relation to student preferences and their levels of competency with regard to the new technology. According to Phillips (23 April 2015 posting by M Phillips to Edutopia, see ‘Notes’), the value of including the students’ voice in decision-making is that it teaches students democracy, helps them develop student leadership, increases student achievement and engagement, and increases the quality of decision-making, as the students add a different perspective than the academics.

The way in which students experience the use of an online program such as Vetbox is an important consideration. According to Law et al. [12], the user experience of products and systems is more personal than social by nature, because it refers to how a user as an individual experiences systems and products. A user’s experience of a particular program is subjective and unique, and not only impacts on his/her willingness to use such products and services immediately, but also on the possibility of prolonged use. In addition, user experience is dynamic by nature and as such it is important to explore user experience prior to developing a product. This is especially needed in education, as education was practised in a static setting for many years [13] and was not necessarily designed to accommodate individual users’ unique experiences.

The OVAH director, who understood the importance of taking the student voice into account [10], met beforehand with various small groups of students and based the initial design of Vetbox on their inputs. However, no explicit follow-up process was in place to evaluate the user experience once the program had been implemented [13].

Although Vetbox was hailed as a revolutionary product that could potentially add tremendous value to the clinical training of veterinary students [9], the student feedback after the initial pilot period was not overwhelmingly favourable. It was soon realised that although the initial student input was used to develop the Vetbox program, the finer details of Vetbox had to be given much more attention if student satisfaction with the product was a desired outcome. In order to refine the program, to get broader buy-in from the student community and to ensure the long-term use of Vetbox, it was necessary to listen even more closely to the voice of the targeted students and to take their full experience into consideration.

## Methodology

Freeman [14] mentioned that although researchers often ask students for their opinion, they seldom include students when developing solutions to the problems students face. Based on the realisation that the student voice had not been sought sufficiently to ensure buy-in from the veterinary students involved in clinical training, their voice was overtly sought by collecting both qualitative and quantitative data from the whole group after the completion of the pilot study. A voluntary group interview (n = 5, purposive sampling) and a structured questionnaire (n = 133) with both closed- and open-ended questions were used (see Appendix). The voluntary group interview was attended by five female students and the intention was to get initial feedback on the students’ experience of using Vetbox to log their procedures. The students were asked to describe their Vetbox experience in general, as well as the specific problems they encountered in using the program. The interview was recorded and transcribed, after which the text was analysed to determine common concerns and recurring trends. The analysis was done according to the method described by Henning, van Rensburg, and Smit [15], through which the process of coding, categorising, and identifying themes results in the identification of recurring trends and patterns. These themes were indicative of the Vetbox issues the students experienced and were therefore transformed into questions that could be used in the questionnaire.

A structured questionnaire was then developed and distributed electronically to the 133 final-year veterinary students who were on clinical rotations at the time. In the questionnaire, students were specifically asked about their Vetbox login experience, and particularly about the process of logging their clinical procedures. Seventy-three of the 133 students, returned the survey, with the majority of them being female (73%). Although some of the students were older than 30 years, most were aged between 25 and 26 years (56%).

For the majority of the questions in the questionnaire a Likert scale response was used that ranged either from *strongly disagree* to *strongly agree*, or from *never* to *always*. In some cases, these questions were followed up with probing open-ended questions. At the end of the questionnaire, four open-ended questions were asked about students’ level of motivation and their recommendations for improving Vetbox for future use. All the open-ended questions were analysed using conceptual content analysis [16], which enabled the research team to derive meaning from those concepts and themes that occurred frequently.

## Results

The results of the student feedback were grouped according to the themes identified in the manner described above. The students’ suggestions were discussed first, and subsequently the underlying student need that was addressed by each of the suggestions was confirmed, using the results of the closed-ended questions and the group interview. Individual student voices were indicated with a unique identifier where direct quotations were used.

### Ease of use and usefulness of Vetbox

**Figure 1.**
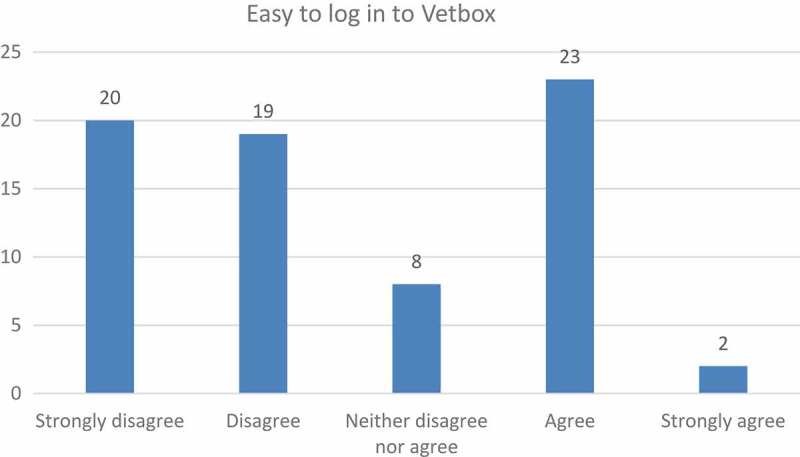
Level of ease of the login process.

Students felt that certain aspects needed to be in place in order for them to use Vetbox. Firstly, the login process needed to be streamlined, the number of actions they had to complete had to be reduced, and the whole experience needed to be made more user-friendly. The students’ frustration was mirrored in the question where nearly half of the students (54%) said that they did not find it easy to log in to Vetbox (Figure 1).

A similar concern was also expressed in the group interview, and in an open-ended item in the questionnaire another student expressed his/her displeasure as follows:

ʽ…the system did not want to accept my username and password on several occasions and after finally being logged on I was kicked off the systemʼ (Participant number 69).

This corresponds to the finding of Davis [17], who points out that students will only use a program if using it is perceived to be free of effort, which, among others, means that the number of clicks has to be limited to a minimum. Students seemed to have experienced the process of logging their procedures in Vetbox as cumbersome and time consuming. For example, one student commented:

ʽ… did try to use vetbox, but after struggling for over an hour on the first two login attempts (each) it was relegated to a waste of time (something not in big supply in final year)ʼ (n = 1).

**Figure 2.**
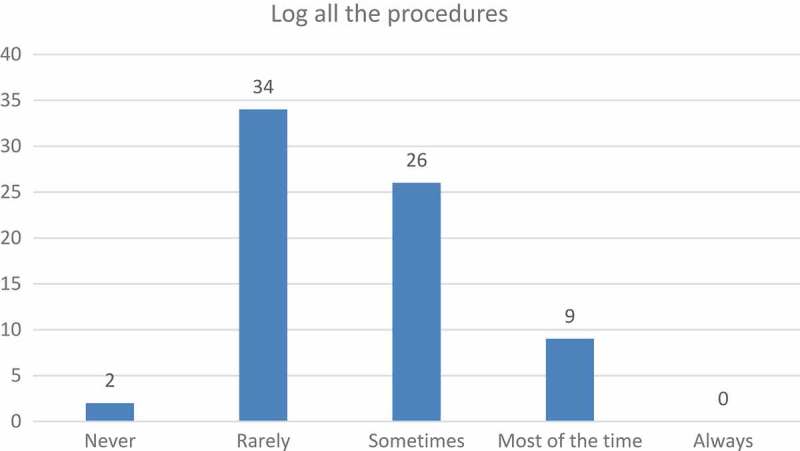
Attempts to log procedures.

When the students were asked whether they attempted to log all their procedures, 51% of the responses were *rarely* or *never* (Figure 2).

In an attempt to suggest ways to increase the number of logged procedures, students recommended that the list of procedures be updated to include some of the more common procedures (like making a blood smear), and that the process to find the specific procedure that they wanted to log be made much easier. For example, they suggested the use of a dropdown list, or autocomplete functionality, to look up a particular procedure. Another suggestion was that if students were busy with, for instance, an equine rotation, the Vetbox program could automatically recommend a list of possible procedures that could be logged when working on horse-related cases, so that students could tick them off as they progressed through the rotation. If they mastered a procedure that was not on the list, then an ‘Other’ comment box could be added, to enable them to enter the information manually. Students also indicated a need to change the number of times they were exposed to a particular procedure, rather than having to add the same procedure repeatedly in relation to different cases.

ʽ…it was not possible to log multiple procedures at once, i.e. performed 50 vaccinations. To log this, every procedure would have to be logged individuallyʼ (n = 26).

**Figure 3.**
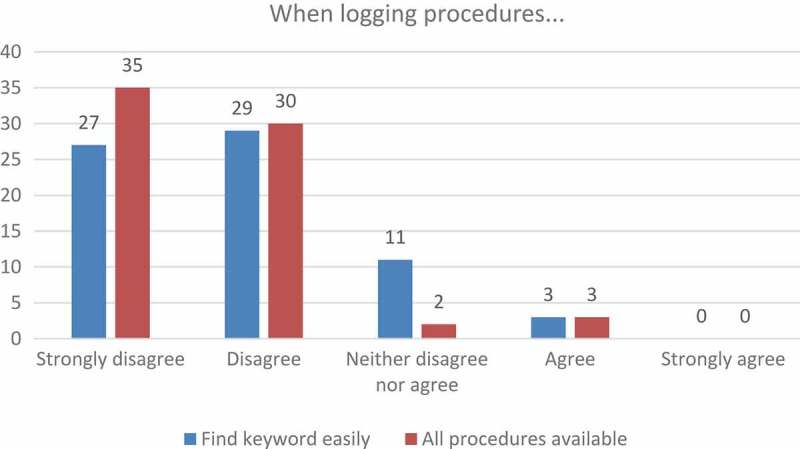
Ease of use relating to the logging of procedures.

This not only correlates with what Davis [17] and Saade and Bahli [18] call perceived ease of use, where the amount of effort needed to operate a system has an influence on whether or not students will use it, and also on the perceived usefulness; whether or not the use of the software would make their lives easier. In this study, the students seemed to have struggled to find the procedure that they wanted to log, and therefore did not experience the program as helpful or as a tool that made their work easier. This struggle to find procedures is reflected in their responses as indicated in Figure 3.

### Motivation to use Vetbox

**Figure 4.**
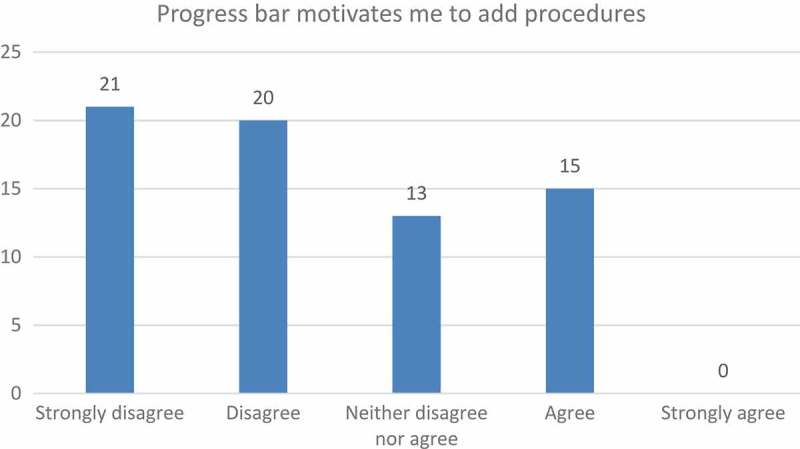
Progress bar as motivator.

The students requested that the way their progress was measured be updated. They complained that even though a progress bar was available, they could not visually see their progress; not even after logging a number of procedures. The perceived slow progress seemed to be unintentionally demotivating. Students’ frustration was reflected in their responses when they were asked if the progress bar motivated them to log their skills (Figure 4).

This led to many of them perceiving themselves as incompetent and slow, whereas a perception of personal competence needs to be present to promote intrinsic motivation [19]. Students were keen to see their own progress after each procedure they logged and seemed to be discouraged when they could not see immediate progress. This is voiced in the following student remark:

ʽ…the progress bar just shows how far you are from finishing, after long hours of filling in the complicated progress of vetbox admin you are disheartened to discover you are only on 3%ʼ (n = 50).

The students experienced this perceived lack of progress as highly demotivating. Malone and Lepper [19], in their taxonomy of intrinsic motivation, refer to four factors; namely challenge, fantasy, curiosity, and control. When discussing ‘challenge’, Malone and Lepper [19] concluded that performance goals should always be personally meaningful before they can be experienced as motivational.

The absence of meaningfulness was also expressed in the comments of the students, as many of them stated that they could not see the value of using both Vetbox and UVIS (Universal Veterinary Information System, a registered trademark, based in Dayton, Ohio, USA) to capture the details of each individual case admitted to the hospital. In addition to the lack of meaningfulness, the much needed feedback from residing clinicians was also lacking. According to a number of researchers, feedback is an important aspect of intrinsic motivation and improved performance [20,21].

Students also felt that they needed an incentive to use Vetbox, as expressed in the next comment:

ʽ…if it was compulsory for our year it would have encourage loggingʼ (n = 59).

This statement by one of the students is in line with the incentive theory of motivation, which proposes that a desire for incentives motivates behaviour [22]. According to Bernstein [23], the incentive theory states that students will be drawn to behaviours that involve positive incentives and will be pushed away from those with negative incentives. It is, however, recognised that these incentives might differ from student to student, and from situation to situation. The fact that students could collate a list of procedures they had been exposed to did not seem to be enough motivation for them to invest time in that task. They stated that they would be more likely to log their procedures if other assessments like portfolios and tests were reduced. A few of them believed that if a mark were allocated for the procedures logged, if they were continually assessed on their progress, or if a clinician were to evaluate them and give them feedback on their mastery of particular procedures, it would in fact have motivated them to keep on using Vetbox. Others felt that incentives could also have included the offer of an exclusive learning opportunity, for example, at a private veterinary practice or attending an unusual surgical procedure.

### Mobility and accessibility

The students suggested that when more than one software program is used, those programs be integrated and synergised in order to make their life easier. In this case, students felt that if Vetbox were linked to UVIS, and all the duplication, such as entering the same case number, diagnosis and treatment plan twice, were removed, they would be more willing to log their cases regularly, as it would take up less of their valuable time.

ʽ…would really save time if UVIS and Vetbox could be linkedʼ (n = 16).

One way to seamlessly integrate Vetbox and UVIS would be if the students could use their mobile device (for example, a smartphone) to scan the code from the UVIS sticker (using a barcode reader or QR code) where all the patient information is already embedded. If all these details were then automatically exported to Vetbox, students would regard it as a significant improvement.

Further suggestions were made with regard to the devices used to access Vetbox. For example, students suggested that Vetbox be designed as a mobile application, where procedures can be logged immediately and not at the end when everybody has to compete for one of the few available computers in the hospital. In a case where procedures are not logged immediately, they suggest that an improved Vetbox application be developed that could be used offline and which would then automatically upload their logs when they next connect to WIFI. While Vetbox were available on the web browser and could be accessed on a mobile device, the user interface and caching abilities were not developed explicitly for use on mobile devices. The alternative to a mobile application was that more computers should be made available at the hospital.

**Figure 5.**
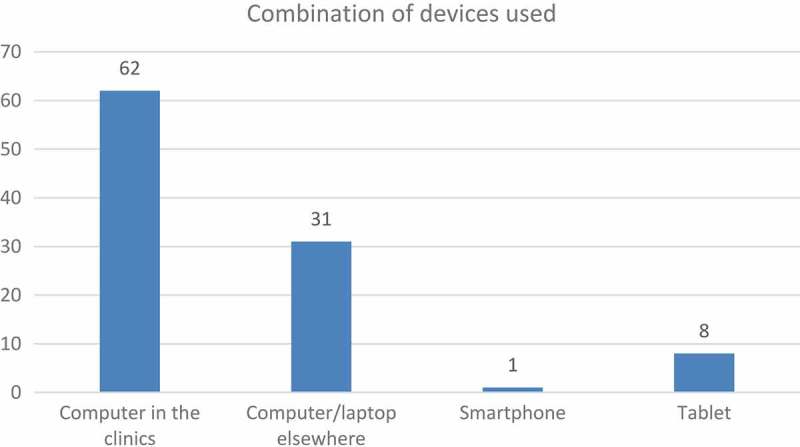
Devices used to log procedures.

In Figure 5, the device distribution is indicated. It is noteworthy to mention that the students indicated that they very seldom used a single device. There was a distinct preference for using a combination of devices, depending on availability and the specific context they found themselves in.

Although the majority of students (91%) opted to use the desktop computers that were available in the clinics or elsewhere on campus, three students indicated that they managed to use only a tablet to capture their work. Even though the number is small, this result serves as an indication that it was indeed possible to log cases using a mobile device, and therefore the possibility of using a mobile device in the clinical environment should be explored in more depth. However, some students mentioned that there might be clinical settings where it would simply not be feasible, or practical, to use an expensive device such as a smartphone or a tablet, for example, in wet labs or in the anatomy hall during a dissection practical. In such cases, the procedures would have to be logged at another location after the session.

The students expressed a need for a dedicated daily time slot during which they could log their cases and procedures, stating that they found it hard to remember details when logging their work at a later stage. The important role of time constraints in the students’ lives is illustrated by the following comment:

ʽ…it was too time consuming and there were too many cases to log, especially during the clinic year, we are well over-worked with long hours and continuous assessmentsʼ (n = 30.

### Training

Lastly, students stressed the need for proper training for both themselves and the clinical staff on the purpose, value, and use of Vetbox. During such training the basic functionalities of the program should be explained. They also indicated that they would appreciate a guide with information on the terminology used in Vetbox to refer to their particular procedures, as many of them struggled to find the specific procedure they wanted to log.

**Figure 6.**
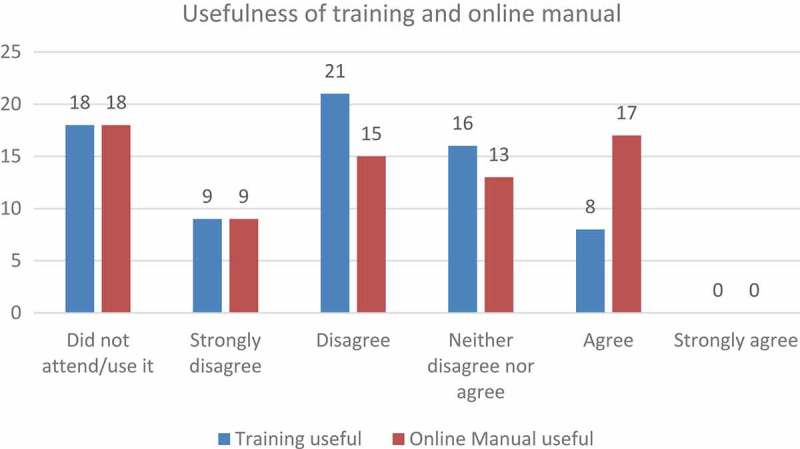
Usefulness of the training session and the online manual.

Despite students emphasising the importance of proper training, 25% of them did not attend the training session that had indeed been offered at the onset of the pilot. Of those who had attended, only 11% found it useful, whereas the rest were either neutral, or did not find it useful at all (64%). Over and above the face-to-face training, an online manual was also made available. Although more students found the manual useful (24%), the majority still did not really experience it as beneficial, despite indicating that they would appreciate a detailed guidebook (Figure 6).

All the students’ comments and suggestions were sent to the hospital director and the software developers so that the necessary changes could be considered for the next version of Vetbox.

## Discussion

Although students’ initial experience of Vetbox was not favourable, they felt that if certain aspects were in place, they would consider using Vetbox. The following is a summary of the students’ feedback in this regard:

- Even though a professional software company developed Vetbox, the user experience was not positive. Students felt that in order for them to consider using Vetbox, the login process needed to be streamlined, the number of actions that they have to complete should be reduced, and the whole experience needed to be made more user-friendly.
- Because of this lengthy process, students avoided logging their procedures. They suggested that the list of procedures be updated and that the lookup procedure be made easier. Students seemed to struggle to find the procedures that they wanted to log, and therefore became discouraged, and did not experience the program as helpful or as an aid that made their work easier.
- Students complained that they could not see their progress on the progress bar while they logged their procedures. This led to a feeling of failure that demotivated them to use Vetbox. However, they wanted to bargain with the researchers and said they would be prepared to use Vetbox if they were to receive incentives.
- The students suggested that when more than one software program is used, those programs should be seamlessly integrated and synergised.
- With regard to the devices used to access Vetbox, students suggested that Vetbox be redesigned as a mobile application, so that procedures could be logged during a dedicated timeslot each day, immediately after performing the procedure, or whenever they had time to do so at a later stage. In addition, the program also needed an offline functionality.
- The students stressed the need for proper training for both themselves and the clinical staff on the purpose, value, and use of Vetbox so that they could make the best possible use of the program.

Whilst veterinary educators perceive Vetbox to be a program that fills an important gap in current teaching and learning practices; one that has been described as ‘impressive, ground breaking and novel’ [9], the first pilot of the software did not receive the positive response that had been envisaged. That was clearly because the voice of the students who were the intended users and beneficiaries of the program had not been sufficiently heard. This finding of this study confirmed the importance and value of exploring the user experience of the end product. Therefore, educators who wish to design and develop software for their own courses are encouraged to listen to the voice of their students during all the phases of the development process, and to be willing to make significant changes based on the user experience of the intended beneficiaries.

## Conclusion

In this study, various valuable suggestions for the improvement of an education software program arose from the students’ feedback. Whilst these recommendations will be considered in the next version of the program, many of the issues raised could possibly have been addressed earlier on if there had been a way in which the students’ voice could have been heard sooner. Further research is needed to determine whether these proposed changes were indeed implemented and whether their implementation did indeed improve the use of the software.

From this experience it can be concluded that the student voice is not only important in the conceptual phase of designing a program, but that students also need to be consulted on an ongoing basis. Better still, it would make good sense to involve students throughout the design process on all possible levels as active members of the team who work together to develop a solution to a problem [14]. This study further corroborates what Phillips (23 April 2015 posting by M Phillips to Edutopia, see ‘Notes’) claims, namely that academics contradict themselves when they say that most changes in education are made for the sake of students, if, in actual fact, the students are not included or have no voice in decision-making. The necessity of continuous inputs by students thus needs to be emphasised.

The participation of students in the development of software programs aimed at improving their learning experience is not only needed for the fine-tuning of the intended software, but it is also of the utmost importance for ensuring the buy-in of the students targeted to use the software. Academics often express frustration when they spend time and energy to develop software for teaching and learning purposes, only to find that the students do not want to use it. Therefore, the importance of taking into consideration the student voice when developing software for students cannot be emphasised enough. When students’ inputs and suggestions are explicitly sought on an ongoing basis throughout the process, from concept development to the ultimate implementation, their specific needs can already be identified and addressed in the early versions of the program. Therefore the student voice is not only important for the design and development of the software, but also for ensuring that students make full use of the program when it is implemented.

## Appendix

### Vetbox Experience Survey

**1. Please indicate your gender (this is only for statistical purposes).**

Female

Male

Other

**2. Please indicate your race (this is only for statistical purposes).**

Black

Coloured

Indian

White

Do not want to disclose

Other (please specify)

**3. Please indicate your age in years at your last birthday.**

**I found the face-to-face VetBox training during our clinic orientation week useful.**

Did not attend it

Strongly disagree

Disagree

Neither disagree nor agree

Agree

Strongly agree

**5. I found the online manual useful.**

Did not use it

Strongly disagree

Disagree

Neither disagree nor agree

Agree

Strongly agree

**6. To access VetBox, I used**:

Computer in clinics

Computer/laptop elsewhere

Smartphone

Tablet

**7. I access Vetbox using**:

My own data bundle

My own WIFI

WIFI on campus

The computer lab connected to UP network

**8. I found it easy to log into Vetbox**

Strongly disagree

Disagree

Neither disagree nor agree

Agree

Strongly agree

**9. Please help us understand why you selected the above answer.**

**10. I attempted to log all the procedures that I completed.**

Never

Rarely

Sometimes

Most of the time

Always

**11. Please help us understand why you selected the above answer.**

**12. It was easy to find the keyword for the procedure I wanted to log.**

Strongly disagree

Disagree

Neither disagree nor agree

Agree

Strongly agree

**13. All the procedures that I wanted to log were available.**

Strongly disagree

Disagree

Neither disagree nor agree

Agree

Strongly agree

**14. Please help us understand why you selected the above answer.**

**15. I logged most of the procedures**:

in the clinic, immediately after completing the procedure.

At the end of the day.

At the end of the rotation.

Only when reminded to do so.

I did not log procedures.

Please explain you answer above and mention challenges you have experienced.

**16. I would prefer to log my cases and procedures as follows**:

UVIS only

Vetbox only

UVIS and Vetbox

UVIS and Vetbox where UVIS and Vetbox are seamlessly linked to one another

Neither UVIS nor Vetbox

**17. Clinicians verified all my procedures.**

Never

Rarely

Sometimes

Most of the time

Always

**18. I would prefer it if the following people could verify my procedures**

Final year students

Nurses

Clinicians

Private vets

Other (please specify)

**19. I would prefer it if the following people could assess my procedures**

Nurses

Clinicians

Other (please specify)

**20. The progress bar motivated me to add all my procedures.**

Strongly disagree

Disagree

Neither disagree nor agree

Agree

Strongly agree

**21. Please help us understand why you selected the above answer.**

**22. I used the feedback option to voice my opinion.**

Never

Rarely

Sometimes

Most of the time

Always

**23. I received regular feedback about my progress from the clinicians through Vetbox.**

Never

Rarely

Sometimes

Most of the time

Always

**24. It is important to reflect on each case/procedure that I recorded.**

Strongly disagree

Disagree

Neither disagree nor agree

Agree

Strongly agree

**25. I could use Vetbox to reflect on my learning.**

Strongly disagree

Disagree

Neither disagree nor agree

Agree

Strongly agree

Please explain you answer above.

**26. I could use UVIS to reflect on my learning.**

Strongly disagree

Disagree

Neither disagree nor agree

Agree

Strongly agree

Please explain your answer above.

**27. In your opinion, why do you think the Faculty introduced the Vetbox system?**

**28. What will encourage you to log your procedures/skills on a regular basis?**

**29. Do you have any suggestions for improving Vetbox as a program?**

**30. Do you have any suggestions for improving Vetbox for logging procedures/skills?**
